# A Dark Side of Telework: A Social Comparison-Based Study from the Perspective of Office Workers

**DOI:** 10.1007/s12599-022-00758-8

**Published:** 2022-07-01

**Authors:** Christian Maier, Sven Laumer, Tim Weitzel

**Affiliations:** 1 University of Bamberg, Bamberg, Germany; 2 Friedrich-Alexander Universität Erlangen-Nürnberg, Nuremberg, Germany

**Keywords:** Social comparison theory, Telework, Envy, Turnover, Job performance, Empirical study, COVID-19

## Abstract

Telework became a necessary work arrangement during the global COVID-19 pandemic. However, practical evidence even before the pandemic also suggests that telework can adversely affect teleworkers’ colleagues working in the office. Those regular office workers may experience negative emotions such as envy which, in turn, can impact work performance and turnover intention. In order to assess the adverse effects of telework on regular office workers, the study applies social comparison theory and suggests telework disparity as a new theoretical concept. From the perspective of regular office workers, perceived telework disparity is the extent to which they compare their office working situation with their colleagues’ teleworking situation and conclude that their teleworking colleagues are slightly better off than themselves. Based on social comparison theory, a model of how perceived disparity associated with telework causes negative emotions and adverse behaviors among regular office workers was developed. The data were collected in one organization with telework arrangements (N = 269). The results show that perceived telework disparity from the perspective of regular office workers increases their feelings of envy toward teleworkers and their job dissatisfaction, which is associated with higher turnover intentions and worse job performance. This study contributes to telework research by revealing a dark side of telework by conceptualizing telework disparity and its negative consequences for employees and organizations. For practice, the paper recommends making telework practices and policies as transparent as possible to realize the maximum benefits of telework.

**Supplementary Information:**

The online version contains supplementary material available at 10.1007/s12599-022-00758-8.

## Introduction

In the 1970s, many organizations started to offer their employees the option of teleworking. This work arrangement allows employees to use information and communication technologies to work at home or another remote workplace (Telework.gov 2010). Telework grew omnipresent in response to the global COVID-19 pandemic as governments forced many organizations to mandate that their employees work from home to the greatest extent possible (Carillo et al. 2020; Chong et al. 2020; Waizenegger et al. 2020). Some scholars expect telework levels in some industries to remain high even after the COVID-19 pandemic is under control (Hafermalz and Riemer 2020). Before the COVID-19 pandemic, many organizations offered telework in response to their employees’ demands for improved work-life balance (Maruyama et al. 2009). Many organizations benefited from telework as well, for example, by saving on average $2,000 per year per teleworker (Howington 2016) due to lower office space costs.

Nevertheless, there were already some practical indications that telework might have a dark side. Several companies such as IBM, Yahoo, Bank of America, Best Buy, and Aetna stopped offering telework before the COVID-19 pandemic (Spector 2017; Wright 2017). They aimed to avoid adverse effects, such as increased turnover and lower performance among employees who work exclusively in the office and have teleworkers as colleagues. This paper refers to such employees as *regular office workers*. Initial anecdotal evidence indicated that regular office workers might be envious of the teleworker colleagues (Odenwald 2017) and experience greater job dissatisfaction because of their colleagues’ teleworking (Wright 2017). It was thought that regular office workers suspected that their teleworking colleagues were entertaining themselves, relaxing at home, or taking care of their family at the expense of getting their work done. In other words, regular office workers may compare their office working situation with their colleagues’ teleworking situation. When evaluating this negatively, they potentially perceive envy or job dissatisfaction, which may, in turn, lead regular office workers to be less productive and even develop intentions to quit their job.

Unfortunately, however, the theoretical and empirical basis for such concerns is limited. Whether and how telework influences regular office workers adversely in terms of increased turnover or decreased performance remains speculative and anecdotal. Nevertheless, this challenge was present before the COVID-19 pandemic and has increased during it. While media, government officials, or other institutions mainly focus on the benefits of working from home and discussing telework as an essential work arrangement for the future, the ones remaining or having to remain in the office tend to be left unnoticed more than ever. Hence, to understand how to implement telework work arrangements successfully and avoid adverse emotional reactions such as envy and job dissatisfaction, it is vital to investigate the adverse effects of teleworking on regular office workers.

We aim to provide a theory on how telework influences regular office workers when comparing their working situation with teleworkers’ working situation. While some teleworkers do all of their work remotely, most teleworkers only do a percentage of their work remotely. In this study, we differentiate between workers who telework at least part-time, whom we refer to here as *teleworkers*, and workers who do not telework at all, whom we refer to here as *regular office workers*. This study asks:

What challenges are generated when regular office workers compare their working situation with that of teleworkers, and how does such comparison affect regular office workers emotionally and behaviorally?

To answer this research question, we take the perspective of regular office workers with colleagues who telework and draw on social comparison theory (Festinger 1954). In this scenario, we propose the concept of telework disparity. We define perceived telework disparity as a contrast-oriented, upward comparison of a regular office worker with their colleagues’ telework. The upward comparison characteristic means that regular office workers compare themselves with others they consider slightly better off than themselves. The contrast-oriented characteristic indicates that comparison-induced negative evaluations may result in adverse reactions. On the one hand, it models that regular office workers perceive a high level of monodirectional, telework-related disparity when they perceive that their colleagues’ working situation is better than their own as they telework. On the other hand, it models no telework-related disparity when they perceive that their teleworker colleagues’ working situation is not better or more beneficial than their own because they telework. Thus, our proposed concept of telework disparity excludes possible perceived disparity in the opposite direction or for other reasons. Our model proposes that perceived telework-related disparity is a potential source of negative emotions, such as envy and job dissatisfaction, and negative behaviors, such as higher turnover intentions and lower job performance. To evaluate the proposed research model and test for the consequences of perceived telework disparity among regular office workers, we use data collected before the COVID-19 pandemic in one organization (N = 269) and take a covariance-based structural equation modeling approach (CBSEM). Our findings confirm our hypotheses and reveal the mediating effect of perceived disparity through envy and job dissatisfaction on turnover intentions and job performance. The importance of the studied topic is confirmed in an applicability check via interviews with regular office workers, teleworkers, and company representatives conducted before and since the outbreak of the COVID-19 pandemic. This study contributes theoretically by introducing and theorizing telework disparity, identifying a dark side of telework, studying telework from the perspective of regular office workers, and explaining how telework can influence turnover intentions and job performance among regular office workers. These contributions are essential when organizations want to offer telework as a work arrangement regularly.

## Literature on Telework and Related Research Gaps

Telework characterizes “moving the work to the workers instead of moving the workers to work” (Nilles 1998). It is defined by the European Union “as a form of organizing and/or performing work, using information technology, in the context of an employment contract/relationship, where work, which could also be performed at the employer’s premises, is carried out away from those premises on a regular basis”. Telework gives employees increased flexibility by considering work as something employees do rather than a place to go to do work. The increased diffusion of communications and secure server technology enables employees to work at home while keeping productivity high (Cameron and Webster 2013).

Most research on telework (Blount 2015; Raghuram et al. 2010) focuses on the benefits of teleworking (for a detailed review on IS-related research in that stream, see Online Appendix C, available online via http://link.springer.com). Research posits that telework is generally good for society by increasing employability and saving energy without impairing the economy (Sharit et al. 2009). From an employee perspective, it has been shown that teleworkers can be more satisfied than regular office workers (Morganson et al. 2010) or can more flexibly respond to family demands (Golden et al. 2006). Telework is a recovery mechanism for teleworkers themselves, as it decreases work exhaustion (Golden 2006). A meta-analysis has concluded that telework has primarily positive consequences for teleworkers (Gajendran and Harrison 2007). While research into such positive effects is dominant, this research has recently been complemented by findings that telework also has adverse consequences. For example, research finds that telework can adversely affect career advancement (Gajendran and Harrison 2007) and increase social and professional isolation (Mulki and Jaramillo 2011) through a lack of social contacts with colleagues (Bloom et al. 2015). Furthermore, previous research has extensively studied telework from a perspective that focuses on the conditions that influence perceptions of telework and the individual consequences of telework. The possible effects of telework on regular office workers, in contrast, have received less research attention. In this stream, we found two papers. First, Ojala et al. (2014) find that telework negatively affects the family and causes stress for family members. Second, Golden (2007) finds that the way regular office workers view their teleworker colleagues *might* be adversely impacted and that this *might* lead them to think about quitting their job.

Our review of relevant literature reveals several research gaps. First, there is only a limited understanding of telework from the regular office worker’s perspective. This study focuses narrowly on whether and how the emotions and behaviors of regular office workers are affected by teleworkers. Second, most scholarship has focused on the benefits of teleworking, largely neglecting its potential *adverse consequences* on regular office workers, such as increased turnover or reduced performance. Interestingly, some large companies had stopped their telework initiatives before the COVID-19 pandemic forced them to let their employees work from home again. They feared negative impacts (Spector 2017), such as that telework might lead to increased turnover intentions or lower job performance among regular office workers. We aim to close these research gaps by applying social comparison theory, developing the concept of *perceived telework disparity*, and developing and testing a research model of its emotional and behavioral consequences on regular office workers.

## Theoretical Background: Social Comparison Theory

Individuals have the inherent tendency to compare themselves with others for self-evaluation and uncertainty reduction (Festinger 1954; Wood 2016). Since this study aims to understand how perceived disparity related to telework influences regular office workers’ emotions and behavior, we chose social comparison theory (Festinger 1954). We follow the recommendation of Truex et al. (2006) to adapt theories of other fields to IS research and take a nominal perspective on the IT artifact. Social comparison theory has two central tenets. First, individuals tend to compare themselves with others they consider slightly better off than themselves, reflecting an ‘*upward drive*’ (Buunk and Gibbons 2007). Second, such upward drive social comparisons affect individuals both positively and negatively. On the positive side, the individual may *assimilate* to the higher standard (Mussweiler and Strack 2000; Pelham and Wachsmuth 1995). On the negative side, *contrast*-oriented comparisons may result in adverse reactions when comparisons reveal negative evaluations (Morse and Gergen 1970). The direction of the comparison depends on the individual’s objectives in terms of self-enhancement or self-improvement (Collins 1996). Self-enhancement objectives are generally associated with downward comparisons (Tesser 1984), resulting in enhanced self-image. Self-improvement objectives are generally associated with upward comparisons, resulting in greater skills and abilities (Taylor et al. 2016). Even though both are possible, comparisons are more often contrast- than assimilation-oriented (Mussweiler and Bodenhausen 2002). As we aim to understand the adverse effects of colleagues doing telework emerging from contrast-oriented comparison, we focus on such contrast-oriented, upward comparisons concerning colleagues who telework. Such contrast-oriented, upward comparisons lead regular office workers to develop a *perceived telework disparity* resulting from social comparisons.

**Fig. 1:**
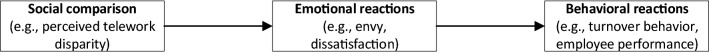
Theoretical perspective on social comparison and its consequences

Such comparison-induced disparities influence the individual in multiple steps (Bamberger and Belogolovsky 2017; Lim and Yang 2015). After evaluating whether they consider someone slightly better off than themselves, individuals experience emotions, which may lead to behaviors (see Fig. 1). Previous research provides strong evidence that the negative emotions *of envy* and *dissatisfaction* are triggered by disparities resulting from contrast-oriented, upward comparisons (Bamberger and Belogolovsky 2017; Myers and Crowther 2009). There is also evidence that such negative emotions may be triggered if an individual, e.g., a regular office worker, evaluates the situation as threatening for themself (Lazarus and Folkman 1984).

Regarding behavioral reactions, research in the strand of social comparison finds that perceived disparity can affect *performance* and *turnover intention* (Hanus and Fox 2015). Since both general management and IS research emphasize the importance of these behaviors (e.g., Bommer et al. 1995; Griffeth et al. 2000; Joseph et al. 2007), this study also focuses on these two behaviors. Figure 1 summarizes our theoretical model, which serves as the foundation for defining the new concept of *perceived telework disparity* and the research model described in the following.

## Conceptualization of Perceived Telework Disparity

For the context of telework, we use social comparison theory (Festinger 1954), positing that employees make contrast-oriented, upward comparisons to show that regular office workers compare themselves with their teleworker colleagues. We focus on regular office workers embedded in a social network of regular office workers and teleworkers. They can easily compare their working situation with the working situation of their teleworking colleagues. Such comparisons happen, and regular office workers might conclude that the working situation for teleworkers is better and/or more beneficial.

We define perceived telework disparity as a contrast-oriented, upward comparison of a regular office worker with their colleagues’ telework. In that definition, the upward comparison refers to the fact that regular office workers compare their working situation with those of teleworkers and consider their teleworking colleagues’ situation better. For instance, they have more time for family or need no travel time. The contrast-orientation characteristic reflects that the regular office worker might develop adverse reactions when the comparison confirms that others are better off. For instance, regular office workers might be less motivated to do their work fast or get envy. So, the narrow definition of telework disparity refers to regular office workers, who do not telework at all, and their perception that their teleworker colleagues are better off than they are. Based on this definition and in line with social comparison theory, perceived telework disparity has two major characteristics through the eyes of a regular office worker. First, the regular office worker has at least one colleague who teleworks at least part-time. Second, regular office workers perceive that their teleworking colleagues are better off than them because they telework. When the regular office worker perceives both characteristics, they may perceive telework disparity.

We conducted in-depth interviews to conceptualize the new construct with its described characteristics.^1^ We interviewed six regular office workers with colleagues who telework at least part-time. The purpose of these interviews was two-fold. First, we aimed to understand the degree to which perceived telework disparity exists. Second, we aimed to discover whether such social comparisons result in emotional and behavioral reactions.

Here are interviewees’ statements concerning telework disparity: “my colleagues who are allowed to work from home have a much easier professional and private life. On the one hand, they do not notice the stress at work and have to take on far fewer administrative tasks on short notice, e.g., when the supervisor simply drops in and distributes tasks. On the other hand, they are more flexible in their private lives and spend much more time with family and friends. For example, I cannot have lunch with my children at noon”. Another interviewee complemented, “I feel like I have to process work for my colleagues”. These exemplary statements illustrate the contrast-oriented, upward comparison characteristic of perceived telework disparity.

Here are examples of statements made by interviewees concerning emotional and behavioral reactions: “I would like nothing better than to be in the situation of my colleagues and also work from home … and this makes me jealous and unhappy”. Another regular office worker said, “it is like a two-tier society, and I wonder if I really want to continue working here”. Similarly, a third interviewee reported, “in the future, I will also not stress myself out and work overtime because of my disadvantaged situation”.

Our interviews confirm that regular officer workers perceive an upward comparison-based disparity between themselves and their teleworker colleagues. The interviews also demonstrate emotional (e.g., envy indicated by feeling jealous and dissatisfaction indicated by feeling unhappy) and behavioral reactions (e.g., turnover intentions indicated by wondering about continuing working here and low job performance indicated by not stressing out and without working overtime) in response to this perceived telework disparity. This definition and initial validity of the new concept is the basis for our research model development.

## Research Model Development

**Fig. 2:**
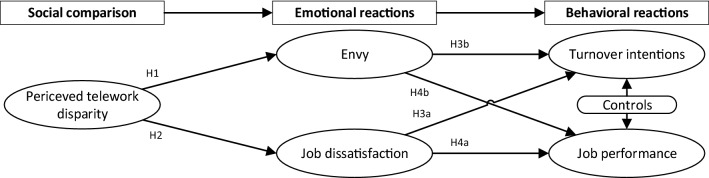
Research model

We take a social comparison theory perspective based on our definition of perceived telework disparity (Fig. 1). We posit that social comparison indicated by relative deprivation influences emotions (e.g., envy, job dissatisfaction) and behavior (e.g., turnover intentions, job performance) (see Fig. 2) as supported by the interviews.

### Emotional Reactions as Response to Perceived Telework Disparity

We start by theorizing that perceived telework disparity causes emotional reactions. Previous research calls it ‘*easy to imagine’* that (upward) social comparisons push envy (Duffy and Shaw 2016). Envy is defined as the degree to which an individual feels that they lack another’s superior quality, achievement, or possession and either desire it or wish that the other person lacked it (Cohen-Charash and Mueller 2007). In the context of telework, we define envy as the degree to which a regular office worker wants to do telework like their colleagues. Even though previous research has not studied whether a relationship between perceived telework disparity and envy exists, similar research has validated that envy on the job results from comparisons in other contexts, including pay disparities (Bamberger and Belogolovsky 2017; Call et al. 2015). In telework, we know that many organizations are highly transparent about telework policies (Hunton and Norman 2010) and employees know which of their colleagues do telework and how much of their work time they do remotely. This transparency might foster envy-producing social comparisons, as regular office workers can easily compare themselves with their teleworker colleagues, which typically takes the form of contrast-oriented, upward comparison (Bamberger and Belogolovsky 2017). When regular office worker compares themselves to someone whose position they perceive as better and more advantageous because they do telework, they may perceive a discrepancy and relative deprivation, possibly at their own expense. This may lead them to perceive envy (Koopman et al. 2019) and react emotionally, such as by wishing that their teleworker colleagues were no longer allowed to telework or that they could telework as well.

The comparison in the telework context, which leads employees to perceive telework disparity, has an even more substantial impact on envy for two reasons. First, when social comparisons are based on objective and concrete facts, which is transparent in the case of telework, individuals react with relatively strong emotions (Kahneman 2012). Second, employees tend to attribute inordinate influence to objective and concrete facts (Tversky and Kahneman 1983), making them unlikely to consider perceived disparities or deprivations as possible errors or distortions and more likely to react with strong emotions. Hence, we hypothesize that:

#### H1

The higher the perceived telework disparity, the higher the envy toward telework colleagues.

Job satisfaction research shows that employees evaluate various job elements (Locke 1969) to determine their job dis/satisfaction. While job satisfaction reflects a positive perception of a job, job dissatisfaction reflects a negative affective orientation toward the job (Vroom 1995). Various job characteristics determine the level of job (dis)satisfaction (Hackman and Oldham 1975). The characteristics of the social network individuals are embedded in are one of them. These social networks include colleagues, supervisors, customers, and many others (Charoensukmongkol et al. 2016). Literature from other streams confirms that such effects between different individuals exist (Stoetzer et al. 2009). For instance, conflicts with patients influence the work-related outcomes of nurses in terms of job dissatisfaction, efficiency, and health (Utriainen and Kyngäs 2009).

We posit an effect of colleagues’ telework on the job dissatisfaction of regular office workers, arguing that the characteristics of the relationship between office workers and their teleworking colleagues determine the level of perceived telework disparity by the office worker. This is based on the fact that much attention has been paid to the potential adverse effects of telework on teleworkers resulting from their relationship itself. Employees consider telework a primary impediment to effective communication, collaboration, teamwork, and work relationships (Grundmann et al. 2011). This includes perceived communication challenges and potentially missed professional opportunities (Bailey and Kurland 2002). Khalifa and Davison (2000) point out that when colleagues do telework, regular office workers are also challenged by communication and group cohesion problems. When regular office workers evaluate work relationships, including communication, collaboration, and teamwork, as worsened by their colleagues’ telework, their level of dissatisfaction increases (Eckhardt et al. 2016). These aspects are triggered by the fact that colleagues’ telework negatively affects employees working at the office, making them dissatisfied with their job. We suppose that these effects are exacerbated, as the regular office workers attribute the job dissatisfaction to the perceived telework disparity, reflecting that their telework colleagues are responsible for that situation and consider themselves worse off. With those theoretical arguments, we hypothesize that:

#### H2

The higher the perceived telework disparity, the higher the job dissatisfaction.

### Behavioral Reactions as Response to Emotions

Behavioral research informs us that emotions are typically not the end-state of an employee’s reaction, as emotions are considered to influence behavior (Zhang 2013). Focusing on envy and job dissatisfaction as two specific emotions triggered by perceived telework disparity, we align with previous research and assume that both influence employee behavior. We concentrate on *turnover intention* and *job performance* as two behavioral reactions. Both are relevant to several different organizational success variables (Huselid 1995). Research in the strand of social comparisons indicates that disparities affect performance and turnover (e.g., Hanus and Fox 2015; Vidyarthi et al. 2010

Turnover intention reflects the deliberate and conscious willingness to quit a job and leave the organization (Tett and Meyer 1993). Research reveals that this willingness is, among others, influenced by emotions such as job satisfaction (Joseph et al. 2007; Maier et al. 2021).

Job satisfaction is the main predictor of employee turnover as theorized and empirically validated by several studies (Griffeth et al. 2000; Joseph et al. 2007; Tett and Meyer 1993). In our context of telework, we argue that perceived telework disparity might change elements of the workplace and thus contribute to job dissatisfaction and potential turnover intention. It has been shown that dissatisfaction can be reduced by acquiring new skills (Lukaszewski et al. 2008) or by adapting one’s behavior (Lazarus and Folkman 1987), which employees may shy away from, reacting instead by increasing their willingness to quit and leave the organization. Envy stemming from perceived telework disparity is an unpleasant and uncomfortable emotion related to physical and psychological pain (Duffy et al. 2012). Envy is a powerful emotion with adverse behavioral reactions (Vecchio 2000). This behavior may include higher turnover intention in the workplace if employees consider quitting an attractive way out of an envy-inducing situation, a specific adaptation mechanism in response to envy (Lazarus and Folkman 1987). Thus, we hypothesize that:

#### H3a

The higher an employee’s job dissatisfaction, the higher the turnover intention.

#### H3b

The higher an employee’s envy, the higher the turnover intention.

We know that job performance, defined as the outcome achieved and accomplished by an employee while working (Anitha 2014), depends on organizational practices, policies, and features. It is central to an organization’s success (Cardy and Leonard 2014). Previous research suggests a large number of aspects as a determinant for job performance, including work engagement (Salanova et al. 2005), organizational climate (Luthans et al. 2008), and job satisfaction (Mobley 1977). We propose a negative relationship between job dissatisfaction and job performance. In telework, when employees perceive telework disparity, they feel less satisfied with their job and less motivated (Locke and Latham 2004) to engage in their work or go the extra mile, which may lead to lower job performance.

Beyond that, envy is a poison in the working atmosphere (Perini 2014), reducing commitment to supervisors and colleagues and ultimately impairing job performance (Becker et al. 1996). When employees perceive telework disparity and envy, they grow less committed, and their job performance deteriorates. Therefore, we hypothesize that:

#### H4a

The higher an employee’s job dissatisfaction, the lower their job performance.

#### H4b

The higher an employee’s envy, the lower their job performance.

Controls. To control our research results, we also respect the influence of alternative theoretical explanations for turnover intention and job performance. We include the control variables age, gender, profession, and trait social comparison orientation, reflecting a dispositional tendency of some individuals experiencing chronic uncertainty concerning the self about others (Eckhardt et al. 2016; Joseph et al. 2007; Thau et al. 2007).

## Research Method and Results

### Procedure and Sample

The study was conducted before the COVID-19 pandemic began. We focused on a single organization to ensure similar environmental conditions among survey participants. We chose an organization in the German automotive industry that employed about 100,000 people and had a sales volume of multiple billion euros at the time of our survey. It had permitted remote work for the past several years, so employees had experiences with and opinions about telework and colleagues doing telework. We ensured that, among others, vacation days were identical for teleworkers and regular office workers in the organization. The organization defined specific criteria for a telework option, including job position. Typically, about 66 percent of the organization’s teleworkers work 2–3 days from home.

The organization arbitrarily selected 300 regular office workers whose job positions did not qualify them for telework, who worked in the office, and who had teleworker colleagues with different job profiles and positions. With top management support, 269 (i.e., approximately 90%) of the selected 300 regular office workers completed the survey within two weeks (see Table 1). C-level managers in the organization reported that about two-thirds of the teleworkers in the organization worked two or three days per week, and all of them worked at least one day per week from home at the time of our survey.

**Table 1:** Demographics of the participants (N = 269)

Demographics (in %)					
Sex	48.7 female,		Profession	IT professional	26.8
	51.3 male			other	73.2
Age (mean 37.4)	< 20	1.1	Work experience (mean 15.9 years)	< 5	25.8
	20–29	34.2		5–9	14.6
	30–39	36.9		10–14	20.8
	40–49	21.5		15–19	17.0
	50–59	4.4		> 19	21.8
	> 59	1.9			

Before conducting the survey, we met with company representatives and the works council to discuss how to preserve the anonymity of survey participants, the survey items, and the organizational specifics.

### Survey Instrument Including Scale Development

We used and adapted measurement scales from well-established and reliable research instruments (see Online Appendix A, Table 4). The dependent variable *turnover intention* was measured with three items (Maier et al. 2013; Thatcher et al. 2002), including ‘I intend to quit my actual job’. *Job performance*, the second dependent variable, was measured with four items (Belanger et al. 2001), including ‘My work environment allows me to do high-quality work’. The mediating variables *envy* (Bamberger and Belogolovsky 2017; van de Ven et al. 2009) and *job dissatisfaction* (Belanger et al. 2001; Maier et al. 2013) were measured with three questions. For instance, ‘I hope that co-workers doing telework would fail at something’ or ‘Overall, I am dissatisfied with my job’. We focused specifically on the malicious quality of envy (Duffy et al. 2012). We based on job satisfaction scales and replaced the term satisfaction with dissatisfaction in each measure for job dissatisfaction.

Since no items to measure *perceived telework disparity* exist, we developed new items. We followed the steps Ragu-Nathan et al. (2008) outlined and developed a pool of items in line with our definition and based on measures studying dispersion in other related research strands (Sen et al. 2006; Suh et al. 2011). We then discussed these in our research team and with regular office workers to ensure content validity (MacKenzie et al. 2011). We incorporated that feedback to redefine the developed items and validated them using the q-sort method (Nahm et al. 2002). In our case, we invited 97 students with work experience from our department to sort our redefined items according to how well they match the construct’s definition. As recommended, we removed three items assigned correctly by less than 61% of the respondents (Nahm et al. 2002) (Table 2).

**Table 2:** Results of q-sorting method to assess the reliability and construct validity

Redefined item	Perceived telework disparity (%)	Other or no assignment (%)
One or more of my colleagues are better off with doing telework more often than I do	**92.78**	7.22
My colleagues are better off with spending a lot of more time working at home than I do	**90.72**	9.28
I am worse off with spending a higher fraction of my overall working time doing telework than many colleagues at my firm. (*reverse coded*) (*removed*)	55.66	44.34
I am worse off with doing less telework than some of my colleagues do. (*reverse coded*)	**91.75**	8.25

Bold means that the values are correctly assigned

Afterward, we collected data via MTurk. The survey participants completed measurement items of perceived telework disparity, envy, IT dissatisfaction, intention to work from home, and telework-related job performance. As recommended in previous literature (Ragu-Nathan et al. 2008), we initially split the data from the 625 respondents into two data sets, performing an exploratory factor analysis on a first set of 375 cases and then validating the results using the remaining 250 cases. Our results show that the three items of perceived telework disparity fall on one factor. We complemented these results with a confirmatory factor analysis showing no high correlations in the error terms of perceived telework disparity, e.g., those for well-established constructs such as IT dissatisfaction were higher. All results were confirmed when we used the second data set. We then tested construct reliability using the combined data sample, calculating a Cronbach’s alpha above the recommended value of 0.7 (Nunnally and Bernstein 1994). When evaluating the whole research model (Table 3), the discriminant validity was examined with the data collected to evaluate the research model. Results show that all indices (e.g., GFI, NFI) are acceptable.

**Table 3:** Descriptive statistics, reliability scores, and inter-construct correlations

Construct		Mean	Std	CR	AVE	1	2	3	4	5	6	7	8	9
1	TSCO	4.44	1.50	0.91	0.72	0.85								
2	Age	See Table 1: Demographics		1.00	1.00	− 0.08	1							
3	Sex			1.00	1.00	0.13	0.04	1						
4	Profession			1.00	1.00	− 0.13	0.02	− 0.22	1					
5	JobDis	3.31	1.34	0.88	0.72	0.02	− 0.13	− 0.14	0.03	0.85				
6	TInt	3.28	1.48	0.92	0.80	− 0.09	− 0.07	− 0.13	− 0.03	0.66	0.90			
7	JPerf	5.03	1.11	0.90	0.70	0.04	0.00	0.07	0.01	− 0.61	− 0.56	0.84		
8	Envy	4.07	1.45	0.89	0.73	0.01	− 0.09	− 0.09	− 0.09	0.43	0.45	− 0.38	0.85	
9	PTD	5.28	1.41	0.91	0.78	0.09	− 0.10	− 0.13	0.06	0.31	0.25	− 0.16	0.49	0.88

The first four constructs are controls, with the *TSCO* trait social comparison orientation, *JobDis* job dissatisfaction, *Tint* turnover intention, *JPerf* job performance, *PTD* perceived telework disparity

We used Likert scales with 1 indicating strongly disagree and 7 indicating strongly agree

Our *control variables* included age, gender, profession, and the trait social comparison orientation, measured with six items (Thau et al. 2007; Zhou and Soman 2003). An exemplary item of that trait is ‘I am not the type of person who compares often with others’.

The survey was conducted in German. We used an iterative process of forward and backward translation (forward translation; discussion with two experts; back-translation; pre-testing the measures with four students and interviewing five additional students to get further insights; final set of measures, which are cross-checked and revised by one native-speaker and one professional translater.

### Preliminary Analyses

This section demonstrates that common method bias, non-response bias, and attrition bias are not an issue in our study. Our sample size is large enough to evaluate the research model (see Online Appendix B). We assessed the viability of the measurement instruments with correlations and reliability measures (Table 3). All that shows that the constructs were internally consistent, have a good convergent validity (composite reliability > 0.7, AVE > 0.5), and have a reasonable discriminant validity (square root of AVE are listed on the diagonal of inter-construct correlations for each construct in Table 3 and these values are higher than the correlations of the construct with other constructs).

### Research Model Parameter Estimation

**Fig. 3:**
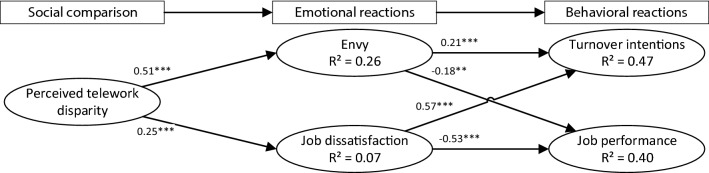
Research results (Note: **P < 0.01; ***P < 0.001). Initially, we included age, gender, profession and the trait social comparison orientation as control variables in our research model. As these do not influence the variables included in the research model, indicated by non-significant relationships, we removed them for the sake of parsimony

We estimated our research model’s statistical parameters by applying the CBSEM technique using AMOS 25 software. We initially included our control variables regarding age, gender, profession, and trait social comparison orientation. While these constructs do not influence the variables included in the research model, indicated by non-significant relationships, we removed them for the sake of parsimony. We then calculated fit across multiple indices.,^2^ which let us conclude that the model has a good fit (χ^2^/df = 1.97; GFI = 0.93; NFI = 0.95; CFI = 0.98; SRMR = 0.052; IFI = 0.98; RFI = 0.94; TLI = 0.97; RMSEA = 0.05). All loadings were over 0.707 and significant (at p < 0.001, see Table 4 in the Online Appendix A). All hypotheses included in the research model (Browne and Cudeck 1993) were significant and in the hypothesized direction. We also see that perceived telework disparity explains 26% of the variance in envy and 7% of the variance in job dissatisfaction, with both explaining 47% of the variance in turnover intentions and 40% of the variance in job performance (Fig. 3).

### Additional Analysis

In addition to our primary analysis, we conducted further analysis to understand the underlying processes better.^3^ We changed our research model slightly to consider a relationship between envy and job dissatisfaction because they are correlated. We suppose this is necessary as the R^2^ value of job dissatisfaction is relatively low when we only consider perceived telework disparity as antecedent. First, we analyzed whether perceived telework disparity has a serially mediating impact on turnover intention and job performance. Second, we changed the initial research model by including the trait social comparison orientation as moderating variable to analyze whether individuals transfer perceived telework disparity differently to envy and job dissatisfaction. We performed a bootstrapping procedure (Hayes 2018) and present the results in the following.

First, to analyze whether perceived telework disparity has a serially mediating impact on turnover intention and job performance, we used PROCESS Model 6 with envy as M1 and job dissatisfaction as M2. For turnover intention, we see a significant positive indirect effect of perceived telework disparity via envy and job dissatisfaction (effect = 0.23; BootULCI = 0.31 and BootLLCi = 0.15). For job performance, we see a significant negative indirect effect of perceived telework disparity via envy and job dissatisfaction (effect =  − 0.38; BootULCI = -− 0.28 and BootLLCi = − 0.48.

Second, we see that the trait social comparison orientation, which we initially included as a control variable in our model, did not influence the variables in the research model. However, we checked whether this predisposition impacts perceived telework disparity’s influence on envy or job dissatisfaction. To analyze this, we performed a bootstrapping procedure (Hayes 2018) using PROCESS Model 84 with trait social comparison orientation as moderator W and again envy as M1 and job dissatisfaction as M2. Our results show that that the trait social comparison orientation moderates the influence of perceived telework disparity on envy (coeff = 0.13; BootULCI = 0.20 and BootLLCi = 0.06), but has no influence on the relationship between perceived telework disparity and job dissatisfaction (coeff = 0.04; BootULCI = − 0.07 and BootLLCi = 0.14).

## Applicability Check

We undertook two applicability checks with the organization (Rosemann and Vessey 2008); the first before the COVID-19 pandemic and the second during the pandemic. This involved lengthy discussions on the topic of telework and about the relevance of our research results with two company representatives and three regular office workers with colleagues who telework. All participants are employees of the company in which we did the survey. We also interviewed two company representatives working at another organization, with some employees doing telework and others working in the office, to gain other perspectives on the issue beyond the organization in which we collected the empirical data. As recommended by previous research focusing on applicability checks, we discussed our results’ importance, accessibility, and suitability with the practitioners.

### Pre-COVID-19 Pandemic

Regarding the importance of teleworking consequences on non-teleworkers, the company representatives reported confusion about the effects of teleworking and whether they should continue their telework policy. For example, it was stated that ‘*we know little about adverse effects resulting from telework. Even though we see many organizations discontinuing employee-friendly teleworking policies, we need more empirical insights to derive practical recommendations about its utility … So the need for further clarification about that important and ever-present topic is required*’. This is supplemented by employees’ statements revealing that teleworking is controversial among employees, even though it is initially a convincing argument in the job interview. For instance, ‘*teleworking practices encourage bad work ethics …* [as teleworking] *leaves the door open for laziness … and might increase teleworkers’ hourly wage compared to* [regular] *office workers*’. Related to that, the employees confirm that ‘*the research findings reflect my personal situation and experiences in that I am so envious when my colleagues work from home. Whenever I call them, they are out at the home improvement store, with birds’ twittering on their terrace or playing with their kids in the sun. All of that is impossible for me working at the organization … plus I have to pick up tasks they cannot do if they are not at their desk. This makes me feel envious and dissatisfied … and ultimately leads to conversations with my partner about whether it would be better if I worked for another organization*’. Finally, it was reported that the findings are valuable and vital, as the results indicatively show that “*telework influences* [regular] *office workers’ own situation* [comment: due to job dissatisfaction] *and the relationship to colleagues* [comment: due to envy toward colleagues] *… and also that telework influences ‘IF’* [comment: in terms of turnover intention] *and ‘HOW’* [comment: in terms of job performance] *employees are willing to work*”.

Regarding the accessibility of our findings, in terms of comprehensibility and readability, the interview partners reported that they could understand our results, particularly that the adverse effects are associated with the two emotional reactions, envy and job dissatisfaction. It was stated that “*the results indicating a relationship between core aspects are easy to understand*”.

Concerning the suitability of the research model and its findings, the company representatives stated that they are ‘*willing to increase transparency* [comment: concerning telework policies and practices] *to avoid envy among staff, dissatisfied employees, high turnover rates and poor job performance*’. It was also stated that the organization would “*enhance the technological infrastructure to improve communication and collaboration among staff*”. An employee working at the office with several teleworking colleagues added *the following: “Discussions with my colleagues show that the spatial distance caused by teleworking is in itself a challenge … but there might be several team characteristics exacerbating or improving the situation, such as offline or private contacts, a high number of or at least experienced team members, colleagues with homogenous working hours or a closer personality and cultural fit between colleagues*”.

### Work During COVID-19 Pandemic

We performed another applicability check in January 2021 to assess the relevance of the derived results during the COVID-19 pandemic, during which telework gained significant importance.

In terms of the importance of our results, all participants of our second applicability check provide some anecdotal evidence about experiences made with telework arrangements during the COVID-19 pandemic. One put it this way: *“I think envy was even worse. We had some people who were not able to work from home. Those risked their health more than those who worked from home. This created situations we had to deal with”*. Another one pointed out: *“Organizational culture is crucial for successful telework arrangements. We realized a lot of negative effects in 2020. Some people could work from home, and others could not. Especially those in the office got dissatisfied and were heavily concerned about their health, simply because their job did not enable a telework arrangement. One employee complained to me that everyone else is safer than she is, which drives her crazy. She envies those working from home for this advantage.”*

Regarding suitability, a top manager of a software company added: “We are considering continuing with our comprehensive telework policy even after the pandemic. However, we realized that one key success factor is to balance the emotions between those who can work from home and those who cannot. In the last months, our employees who still had to work in the office got more emotionally exhausted because they thought everybody values those working from home and thereby ignored the risks and value provided by those who still had to work from the office. We realized the need to manage this envy now, but also after the pandemic to make telework a success.”

In summary, these interviews provide insights into and anecdotal quotes that show the importance of the consequences of teleworking on non-teleworkers in regular and pandemic-specific telework arrangements. They also confirm the accessibility of our findings and initial suitability towards establishing awareness that others’ telework influences regular office workers.

## Discussion, Contributions, and Implications

This research is motivated by diverse experiences organizations and employees made with telework before the COVID-19 pandemic and the increasingly high popularity of telework during the COVID-19 pandemic. We study effects between regular office workers, defined as employees who work exclusively in the office, and their teleworker colleagues, defined as employees who regularly work at home at least part-time. We follow the central tenets of social comparison theory and adapt them to the context of IS research (Truex et al. 2006) by suggesting that employees tend to compare themselves and their situation with others. Our results with 269 regular office workers confirm that perceived telework disparity leads to increased envy and job dissatisfaction. Perceived telework disparity explains more variance of envy than of job dissatisfaction. Reasons for this difference are speculative. It seems reasonable that disparities have stronger influences on variables with a component in which individuals compare their situation with other people’s situation (e.g., envy). Contrary, other variables closely related to one’s job (e.g., job dissatisfaction) are influenced by a broader range of dimensions (Aziri 2011). With our post-hoc analysis, we carve out that individuals react differently to perceived telework disparity depending on whether they have the personal inclination to compare themselves often with other people. Interestingly, the trait social comparison orientation moderates the influence of perceived telework disparity on envy but not job dissatisfaction. This might be explainable with findings from previous research suggesting that highlights the importance of the trait social comparison orientation for envy (Geng et al. 2021). Contrary to that, the insignificant moderating effect might be explainable, with our post-hoc findings, revealing that job dissatisfaction is itself influenced by envy. We also see in our results that turnover intention and job performance are more firmly grounded in job dissatisfactions than in envy. This finding might be related to the fact that job dissatisfaction is the strongest predictor for turnover intention (Joseph et al. 2007). We next focus on the theoretical contributions and practical implications related to those findings.

### Theoretical Contributions

This study contributes theoretically in several ways.

*Providing Theory for the Perspective of Regular Office Workers on Telework*

Extant research on telework has taken an intrapersonal perspective, focusing solely on the causes and consequences of telework employees (Blount 2015). However, these research approaches fail to address practical observations that telework influences colleagues who do not telework. This study contributes by taking the perspective of regular office workers on telework and providing theoretical arguments to explain how telework can adversely affect regular office workers. In line with social comparison theory, we theorize the concept of perceived telework disparity as a contrast-oriented, upward comparison with colleagues who do telework. With this perspective, we now understand how teleworking influences regular office workers' thoughts and behavior adversely.

*Explaining the Existence of a Dark Side of Telework*

We know from research that telework has many benefits for both employers and employees (Gajendran and Harrison 2007). However, little research has been undertaken about the possible pitfalls and challenges caused by telework. We suggest the concept of perceived disparity between one’s own and one’s colleagues’ extent of telework. Our empirical results indicate that employees experience this disparity, as illustrated by comments made in the applicability check. Using a model based on social comparison theory, we provide theory to explain that not being permitted to telework while colleagues are permitted to telework and do so at least part-time results in envy and job dissatisfaction. We further show that mediated by these emotional responses perceived telework disparity leads to lower job performance and higher intentions to quit. We contribute to the ongoing discussion of whether telework is beneficial for employers and employees with those results. We indicate that offering telework options might be a clear risk for an organization when their regular office worker quits or performs worse due to disparity perceptions, representing a dark side of telework.

*Identifying an Additional Driver of Increased Turnover Intention and Decreased Job Performance*

This study theorizes and provides empirical evidence that perceived telework disparity can lead to intentions to quit and lower job performance among regular office workers. Extant research has attributed these behavioral reactions to job characteristics in general, including role stress and behavior or organizational factors including advancement and rewards (Joseph et al. 2007). This study thus contributes by providing the specific job characteristic of perceived telework disparity as an additional driver of turnover intention and low job performance, mediated by envy and job dissatisfaction. So, even while organizations offer telework to provide employees an ideal working situation, it might have adverse consequences for organizations, such as employee turnover or low job performance.

*Offering Initial Insights into the Importance of Predispositions*

A vast amount of research emphasizes the importance or predispositions to understand emotional and behavioral reactions to certain situations (Pflügner et al. 2021a; 2021b). We provide an explorative explanation for why some regular office workers react with envy when they perceive telework disparity. We find that the predisposition social comparison orientation moderates the development of envy. We see that employees scoring with a high social comparison orientation tend to react to telework disparity with envy. This is important as previous research has not studied which individual differences are more relevant to the development of envy than others. Apart from that, our exploratory results carve out the importance of focusing on predispositions that have a high fit to a specific context (Maier et al. 2019) because, interestingly, the predisposition social comparison orientation has no moderating influence on the relationship between perceived telework disparity on job dissatisfaction. This indicates that the predisposition is only relevant for emotions that focus on comparisons with others, e.g., envy, but not for emotions that focus on one’s working situation, e.g., job dissatisfaction. Overall, the findings emphasize that individuals, who often compare themselves with others, have a more difficult life and perceive more negative emotions such as envy.

*Triangulating Empirical Results within the Applicability Check*

We discussed our results with company representatives and employees. Our interview partners agree that the topic is significant. They report a high uncertainty about the benefits and challenges related to teleworking. The danger of social loafing (Alnuaimi et al. 2010) was mentioned as a possible drawback of telework. Practitioners indicatively note the need to differentiate between different team compositions. For teams with some members who sometimes telework, practitioners of our applicability check underscore the importance of fit among team members and that part-time teleworkers are socially well-embedded with their colleagues (Maier et al. 2021; Rai et al. 2009) and aware of daily office practices. Practitioners report the danger of mutual misunderstanding for teams with full-time teleworkers and regular office workers. Company representatives see the need for research into best practices for reducing perceived disparity and its negative consequences beyond ensuring high transparency. This lets us conclude that the importance of having potentially negative consequences in mind is highly relevant when offering teleworking as a perk to attract top talent.

*Perceived Telework Disparity and the COVID-19 Pandemic*

The applicability check conducted during the COVID-19 pandemic also allowed us to discuss our empirical results derived from a pre-COVID-19 study with company representatives and employees in light of the specific experiences made during the pandemic. In this context, our interview partners point out that perceived telework disparity was a significant challenge during the pandemic. Even during the pandemic, when telework was not voluntary but legally mandatory for most employees, some employees had to remain in the office. These employees risked their health even more than those who could work from home. Hence, this specific telework disparity was even more challenging because only a few employees had to work in the office. Accordingly, this provides first insights into the relevance of telework intensity, in terms of both the number of employees teleworking and the number of days employees telework, and the job characteristics for perceived telework disparity: The higher the number of colleagues doing telework, the more time colleagues spend working from home, and the less one’s job profile enables telework, the worse the perceived telework disparity. Thus, perceived telework disparity is relevant when some employees choose to telework, whereas others do not. These insights substantiate that we learned a lot about telework during the COVID-19 pandemic, but telework in the post-COVID-19 era might follow different patterns as work circumstances are different.

### Practical Implications

This study identifies the potentially challenging adverse effects of perceived telework disparity on regular office workers. These challenges were observed before the COVID-19 pandemic (Odenwald 2017; Wright 2017) and reported as necessary during the pandemic. There were still workers who had to remain in the office (Waizenegger et al. 2020), and it is expected to be a challenge after the pandemic again when some employees still have the choice to telework, whereas others do not (Carillo et al. 2020). The implications derived from this study are relevant for organizations regarding how to implement telework work arrangements successfully and help employees avoid or manage adverse emotional reactions to them.

Organizations should resolve perceptions of telework disparity and, second, should make arrangements that might prevent feelings of envy and job dissatisfaction from arising. Our interviews for the applicability check indicate transparency as a key to whether telework disparity negatively affects regular office workers emotionally and behaviorally. We recommend making telework practices and policies as transparent as possible to realize the maximum benefits of telework. To increase transparency, teleworkers should provide their telework schedules and their availability. It was reported that telework disparity, grounded in social comparison, can cause uncertainty. This uncertainty can be exacerbated when organizations do not publish guidelines or leave regular office workers alone to handle the challenges they face when their colleagues telework. Publishing transparent guidelines is thus a first step toward minimizing the potential adverse effects of telework disparity. Setting regular, fixed dates for status updates, especially for teams composed of regular office workers and teleworkers, was also mentioned to reduce envy and job dissatisfaction. A further measure to reduce these perceptions and feelings is to establish mandatory office days to encourage personal contact and exchange.

Organizations should take measures drawing on best practices for managing virtual teams (Staples et al. 2004) to ensure envy-free, close and efficient collaboration between teleworkers and regular office workers. This is confirmed by organizational representatives’ reports that teams with teleworking members are more successful if they include, among others, at least some experienced team members or have similar working hours. In terms of technological infrastructure, possible measures extend beyond connectivity technologies for enablements, such as broadband connectivity, computing mobility, applications, and secure access to data, including collaboration technologies for productivity and effectiveness, such as voice and video conferencing, messaging, and sharing and knowledge tools. Regular office workers also report their willingness to participate in training courses in setting up virtual meetings to avoid wasting valuable time on technicalities.

### Limitations

First, our research focuses on how part-time teleworking influences colleagues who only work at the office. With that, we cannot make statements on how full-time teleworking influences colleagues in the office with whom they share weak or no social relationships or whether also teleworkers perceive a disparity when having colleagues who do more telework than they do. Second, with the study following a cross-sectional design, we cannot make statements about the effects of perceived telework on envy, job performance, or turnover intentions over time. Since we measured job performance using self-assessment, following the lead of some other IS research, we did not consider supervisors’ assessments of employee job performance. Related to that, we evaluated the research model with data collected from one single organization. While we did this to keep the context of surveyed employees stable, the results might be limited as this prevents us to from determining the extent to which the context impacts the results. Thus, it impossible to assess whether the target company provides good or bad, fair or unfair, generous or stingy telework terms and conditions, vis-à-vis regular office workers. Insights on that require additional research that considers contextual factors such as salary, work conditions, duties, and management styles. In addition to that, the focus on one organization does not offer insights on whether regular office worker compares their situation with teleworkers in their organization or whether they have preconceived notions against telework in general that might color their views. In our items we refered to “my colleagues” to refer to people in the organization our survey participants are based in, but we cannot control whether our participants had, in fact, their teleworking colleagues in this organization in mind. Results from our pre-survey and post-survey interviews indicate that people talked about their colleagues in the organization they are based in. Third, we measured job performance with items that focus on the work environment. It might be helpful for additional insights to base on our findings and use different measures that purely focus on job performance without referring to employees’ work environment. Such research might, then, generalize the results without focusing on one organization or a specific work environment. Fourth, the study was conducted before the COVID-19 pandemic. Hence, specific experiences made during the forced telework work arrangement might impact the perceived relationship between teleworkers and office workers, which we cannot control. This leaves the opportunity to replicate our study and approach some of the following opportunities for future research on these effects of telework during the pandemic and afterward to compare the different effects we observed and draw conclusions about the influence of telework experiences during the global COVID-19 pandemic on these effects. Finally, our research focuses on the adverse effects of envy. It leaves room for the possibility that envy could drive positive motivation to improve one’s skills and/or performance to get a position within the organization that is permitted to telework.

### Future Research Directions

**Fig. 4:**
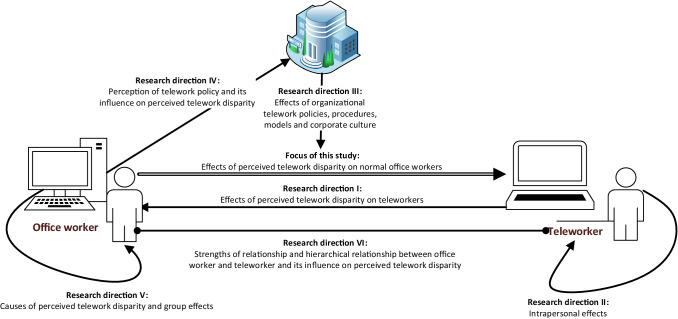
Focus of this study and future research directions needed to provide generalizable findings

This study considers the effects of perceived telework disparity on regular office workers by taking a social comparison theoretical approach. Figure 4 illustrates the focus of this study and six specific potentially fruitful avenues of future research.

I Future research might consider the effects of telework disparity on teleworkers with colleagues who telework more than they do or who telework full-time. More research is needed to understand if, why, and when teleworkers perceive telework disparity and how these perceptions influence their emotions, thoughts, and behaviors. Such research might consider, for instance, perceived social exclusion, perceived disparity in leadership responsibility and professional advancement opportunities. This would complement our study, showing dark sides of teleworking for regular office workers and teleworkers alike.
II A further related avenue may consider the intrapersonal effects of teleworking on teleworkers. Such challenges may arise from blurring the border between work and family issues and increased perceived work-home conflicts. Such challenges have less to do with perceived telework disparity and more to do with conflicting priorities, such as between working and taking care of the family. Being close to the family might intensify adverse feelings or behaviors, such as feeling socially excluded or performing worse.
III A third avenue of potential research could focus on the role of organizational telework policies, procedures and models, and corporate culture on the effects of perceived telework disparity. This avenue of research would require collecting data from multiple organizations with a wide range of telework policies, procedures, and models, focusing on power structures, rewards and perks, trust, and corporate culture. Such research could provide best practice approaches explaining how to implement telework in the corporate culture while avoiding adverse consequences. Future research might develop a multi-level model that illustrates how distinct contextual factors (e.g., management-, organizational-, industry-, and cultural-specific factors) influence employees’ perceptions of telework. Notably, while this research has focused on a dark side topic caused by telework, there are many positive issues for organizations, society, and individuals. For instance, reduced commute time and associated energy use, greater time flexibility, increased productivity due to fewer interruptions, and more efficient use of space and other resources. Future research should broaden the discussion to include the full range of positive and negative aspects of telework to inform management considerations and decisions and organizational models and guidelines.
IV A fourth closely related avenue of research could examine gaps between an organization’s understanding of its telework policies, procedures, models, and corporate culture and the employees’ perception of them. Our interviews revealed significant gaps in understanding and perception. Specifically, our interviews with regular office workers during the applicability check indicatively reveal that most of them were not familiar with or had an incorrect understanding of organizational policies. This underscores the need for best practices in terms of communicating telework policies and procedures, communicating and encouraging adherence to transparency guidelines, and monitoring telework employee behavior to mitigate the potential adverse effects of perceived telework disparity on turnover intention or reduced job performance.
V A fifth avenue of potential research could focus on antecedents of perceived telework disparity. While our study focuses on reactions to perceived telework disparity, it leaves room to study its causes. Research might use appraisal theory (Lazarus and Folkman 1984) and study whether evaluating work conditions as hindering (Maier et al. 2021) is a source of perceived telework disparity. Similarly, future research might analyze group effects on strengthening perceived telework disparity. Such research could take a social contagion perspective and consider whether or how perceived telework disparity and the emotions and behaviors it can trigger are transmitted among regular office workers. Research indicates that individuals imitate observed behaviors or adopt observed emotions (Sun 2013), so emotions and behaviors stemming from perceived telework disparity might be spread among other regular office workers via complaining about teleworkers and perceived telework disparity. Such research would help explain how and why negative perspectives on telework disseminate among regular office workers.
VI Finally, a sixth fruitful area for future research is identifying which and how factors moderate perceived telework disparity. This research considers the effects of perceived telework disparity on emotions as a linear effect. Acknowledging research proposing curvilinear effects (Sun and Zhang 2006), future research might focus on factors including team structures, team interactions, and the social and hierarchical relationships between all team members where telework is involved. Such research might test, for example, whether the adverse effect of perceived telework disparity is larger or smaller if supervisors do more telework than when colleagues of the same hierarchical level do more telework. Similarly, such research compares the effects of perceived telework disparity given strong-tied relationships with the effects of perceived telework disparity given weak-tied relationships or no relationship. Mediating factors in such studies may include leisure time contact and levels of trust.

## Conclusion

Previous telework research has taken an intrapersonal perspective and focused on telework’s implications and benefits. This study identifies the causes of adverse effects of perceived telework disparity. We theorize and empirically validate the concept of perceived telework disparity, which results in envy, job dissatisfaction, turnover intentions, and decreased performance among regular office workers.

## Supplementary Information

Below is the link to the electronic supplementary material.

Supplementary file1 (PDF 996 kb)

## Footnotes

1 We interviewed six regular office workers (three men, three women; the mean age was 43, ranging from 28 to 55). With an interview guideline, we questioned regular office workers about their beliefs, thoughts, and feelings related to their colleagues who work from home at least some of the time. The interviews were conducted by one of the authors and another researcher and lasted between 20 and 35 min. We recorded and transcribed each interview and used these data as the basis for our qualitative analysis (Fielding and Schreier 2001; Yin 2009) using the software program MaxQDA and to develop an understanding of perceived telework disparity (Miles et al. 2013; Myers 2009).

2 Chi square/df represents the minimum discrepancy divided by the degrees of freedom. Goodness-of-Fit Index (GFI) indicates the relative amount of variance and covariance that the model explains. Normal Fit Index (NFI) and Comparative Fit Index (CFI) indicate the percentage enhancement in fit over the baseline model. Standardized Root Mean Square Residual (SRMR) represents the standardized difference between observed and predicted covariance. The incremental index of fit (IFI) is used to address the issue of parsimony and sample size. The Reflexive Fit Index (RFI) is obtained from the NFI. The Tucker-Lewis coefficient (TLI) adjusts NFI for the degrees of freedom and penalizes model complexity. Root mean square error of approximation (RMSEA) is one of the most frequently used measures of goodness-of-fit (Kenny et al. 2014). Prior research suggests that chi square/df values should range between 1.0 and 5.0 (Salisbury et al. 2002; Chin et al. 1997). Appropriate values for GFI should exceed 0.9. NFI values between 0.9 and 0.95 represent an acceptable fit and those exceeding 0.95 indicate a good fit (Salisbury et al. 2002). CFI should be at least 0.9 for a good fit or close to 1 for a very good fit (Salisbury et al. 2002; Bentler and Bonett 1980. For SRMR, values less than 0.08 indicate a good fit (Hu and Bentler 1999). In addition, the values for IFI, RFI and TLI should exceed the threshold of 0.9 (Bollen 1989; Salisbury et al. 2002) and RMSEA values of 0.05 or less are indicative of close fit of the model (Browne and Cudeck 1993).

3 We are grateful for the AE’s recommendation to perform this analysis, which led to valuable insights.
