# Supplementary material for: A Dark Side of Telework: A Social Comparison-Based Study from the Perspective of Office Workers
Source: Bus Inf Syst Eng. 2022 Jul 1;64(6):793–811. doi: 10.1007/s12599-022-00758-8 (PMC9247925; doi:10.1007/s12599-022-00758-8)
Supplement: Supplementary file 1 — Supplementary file1 (PDF 996 kb) [file 12599_2022_758_MOESM1_ESM.pdf]

A Dark Side of Telework: A Social Comparison-Based Study From the Perspective of Office Workers

Christian Maier, Sven Laumer, Tim Weitzel

Business & Information Systems Engineering (2022)

Appendix (available online via http://link.springer.com)

Appendix A: Measurement Items and Loadings

Table 4: Measures and loadings

Construct		Question	Loading
Perceived telework disparity (*self-developed*)	PTD-1	One or more of my colleagues are better off with doing telework more often than I do. [Es gibt Kollegen, die besser gestellt sind als ich, denn sie machen öfters Telearbeit als ich.]	0.76
	PTD-2	My colleagues are better off with spending a lot of more time working at home than I do. [Meine Kollegen sind besser gestellt als ich, denn sie arbeiten mehr von zu Hause aus als ich.]	0.91
	PTD-4	I am worse off with doing less telework than some of my colleagues do. (*reverse coded*) [Ich bin schlechter gestellt als meine Kollegen, da ich weniger Telearbeit mache als einige von ihnen.]	0.98
Note: The following questions refer to your feelings toward working with co-workers doing telework.			
Envy (Bamberger and Belogolovsky 2017; van de Ven et al. 2009)	Envy-1	The experience felt frustrating.	0.73
	Envy-2	I wanted to hurt co-workers doing telework.	0.86
	Envy-3	I hoped that co-workers doing telework would fail at something.	0.95
Job dissatis-faction (Belanger et al. 2001; Maier et al. 2013)	JobDis-1	I feel dissatisfied with the way I work at the moment.	0.87
	JobDis-2	I feel dissatisfied with important aspects of my job.	0.92
	JobDis-3	Overall, I feel dissatisfied with my job.	0.76
Turnover intention (Maier et al. 2013; Thatcher et al. 2002)	Tint-1	I often think about quitting my job at my current employer.	0.88
	Tint-2	I intend to quit my actual job.	0.92
	Tint-3	I think about leaving my actual employer.	0.89
Job performance (Belanger et al. 2001)	Perf-1	My work environment allows me to meet the expectations of my supervisor in performing my job.	0.80
	Perf-2	My work environment allows me to do high-quality work.	0.84
	Perf-3	My work environment allows me to complete work in a timely and effective manner.	0.85
	Perf-4	My work environment allows me to improve my overall work performance.	0.87

Table 5: Results of an exploratory factor analysis (using SPSS)

PTD-1	0.950	0.462	0.280	0.246	-0.133
PTD-2	0.930	0.475	0.256	0.246	-0.193
PTD-4	0.870	0.356	0.217	0.150	-0.076
Envy-1	0.394	0.927	0.309	0.328	-0.342
Envy-2	0.431	0.946	0.354	0.414	-0.318
Envy-3	0.531	0.805	0.453	0.463	-0.373
JobDis-1	0.224	0.295	0.932	0.475	-0.486
JobDis-2	0.289	0.408	0.932	0.580	-0.478
JobDis-3	0.269	0.399	0.805	0.579	-0.539
Tint-1	0.217	0.380	0.572	0.920	-0.489
Tint-2	0.229	0.430	0.539	0.934	-0.480
Tint-3	0.210	0.365	0.526	0.932	-0.467
Perf-1	-0.137	-0.346	-0.395	-0.450	0.859
Perf-2	-0.144	-0.401	-0.513	-0.433	0.884
Perf-3	-0.106	-0.299	-0.478	-0.443	0.896
Perf-4	-0.175	-0.281	-0.521	-0.511	0.885

Appendix B: Common Method Bias, Non-Response Bias, Attrition Bias, and Sample Size Requirements

Empirical research must consider **common method bias** (CMB) in self-reported data (Chin et al. 2012; Podsakoff et al. 2003). We used different techniques to minimize common method concerns in setting up the survey. We ordered the questions randomly, and the order was different for each survey participant. To calculate the extent of CMB, we ran two tests. The first test results, Harman's single factor test, which indicates whether or not the majority of the variance can be explained by one factor, show that only 24 percent of the variance of the data is explained by one factor. Second, we used the procedure of examining the correlation matrix as specified by Pavlou et al. (2003). Extremely high correlations ($r > 0.90$) indicate CMB, but our correlation matrix did not show such high correlations. These results indicate that CMB should not be an issue for the results.

We then tested for **non-response bias** by comparing the sample demographics from Table 1: age, sex, profession, and work experience. We compared these with those who were invited, but did not participate. Using a t-test, we saw no significant differences in either set of comparisons.

Next to talking about non-response bias, we also carried out an **attrition test** to check whether our data sample with its 269 participants is different from those invited (300 individuals) and those invited to do telework in the organization. We again compared age, sex, profession, and work experience and used a t-test. Results show no significant ($p>0.05$) differences between our data sample and the other two data samples, so attrition bias is not an issue for the results.

We also followed Kim's (2005) suggestion that a given level of power depends on a) the number of variables/degrees of freedom, b) the relationship among the variables, c) choice of fit index, and d) the value of the fit index and proposes a range of different fit indexes. Using Steiger's gamma (with $\gamma=0.95$; $\alpha=0.05$; *Power* = 0.90), the proposed sample size is 115 and using RMSEA ($\alpha=0.05$; *Power* = 0.90) reveals a minimum proposed sample size of 205.3 for the proposed research model. The study's sample size, which was 269, meets all of these conditions, so our **sample size** is large enough to estimate the research model.

Appendix C: IS Literature on Telework

The following table presents the IS literature on telework during the pre-COVID-19 era. The literature search was conducted using the scientific database Scopus and the search string "[telework OR "remote work" OR "telecommut*" OR "virtual work"] AND [co-worker OR coworker OR colleague OR "office worker" OR non-teleworker]". We focus on papers published in AIS8 using qualitative or quantitative data from the pre-COVID-19 episode.

Table 6: IS literature on telework

Authors	Summary
**(Belanger et al. 2001)**	Investigates how the availability of information system technology, the availability of communication technologies, and communication patterns of telecommuters within their workgroups impact outcomes in telecommuting.
**(Bélanger and Allport 2008)**	Investigates how improved collaborative technology among a group of knowledge teleworkers impacts their communication patterns. This shows that increased collaborative technologies result in an adjustment between explicit and tacit knowledge sharing, as the availability of a centralized and updateable database reduces workgroup communications.
**(Bernardino et al. 2012)**	Identifies guidelines for implementing and managing telecommuting to overcome the demand for highly skilled professionals for IT service providers. This shows that telework should be based on trustworthy relationships and must be supported by a decentralization strategy of both structure and organizational assets.
**(Duxbury and Neufeld 1999)**	It offers an empirical evaluation of the impacts of telecommuting on intra-organizational communication (superiors, subordinates, colleagues, and clients). Results suggest that part-time telework arrangements have little impact on intra-organizational communication.
**(Fritz et al. 1998)**	It compares individual satisfaction with office communication in telecommuting and conventional work environments.
**(Igbaria and Guimaraes 1999)**	Explores whether turnover intentions and their determinants differ for telecommuters and non-telecommuters. Result: Telecommuters faced less role conflict and role ambiguity, were happier with their supervisors, more committed to their organizations, but less satisfied with their peers and with promotion.
**(Iscan and Naktiyok 2005)**	Examines the effects of demographic characteristics, household attributes, support factors, and perceived advantages and disadvantages of telecommuting to individuals, organizations, and society on individuals' attitudes towards telecommuting
**(Neufeld and Fang 2005)**	Develops a general theoretical framework to understand telecommuter productivity. Results: Telecommuter beliefs, attitudes, and quality of social interactions with managers and family were strongly associated with productivity. Also, their social interactions with colleagues, managers, and family influenced their beliefs and attitudes about telecommuting.
**(Stephens and Szajna 1998)**	It analyses the perceptions, expectations, and drivers behind an employee's decision to choose a telecommuting work arrangement.
**(Suh and Lee 2017)**	The paper develops and tests a theoretical model that predicts a teleworker's technostress and job satisfaction by drawing on the technostress model and job characteristics theory. It helps to understand why some teleworkers enjoy the benefits of telework whereas others experience technology-induced stress.
**(van der Meulen et al. 2019)**	Addresses the interaction effects of media synchronicity and temporal and spatial separation among colleagues on sharing in knowledge networks. Special attention is paid to knowledge awareness and homogeneous and heterogeneous knowledge sources.
**(Yap and Tng 1990)**	It examines the factors associated with the attitudes of female computer professionals in Singapore towards the potential implementation of telecommuting in their organization.
